# The effect of thermal processing on the allergenic activity of peanuts

**DOI:** 10.1186/2045-7022-5-S3-P113

**Published:** 2015-03-30

**Authors:** Rebekah Sayers, Justin Marsh, Aida Semic-Jusufgic, Adnan Custovic, Angela Simpson, Helen Brown, ENC Mills

**Affiliations:** 1 The Institute of Inflammation and Repair, Manchester Institute of Biotechnology, Manchester Academic Health Sciences Centre, University of Manchester, Manchester, UK; 2 Campden BRI (Chipping Campden) Limited - Part of the Campden BRI Group, Chipping Campden, Gloucestershire, UK

## Background

Peanut allergy is considered one of the most prominent food allergies, which often results in severe reactions and can be fatal. It can be attributed to one or more of the 12 recognised peanut allergens, the main peanut allergens being Ara h 1, Ara h 2/6 and Ara h 3. Ara h 2/6 are 2S albumins, sensitisation to which is associated with the most severe allergic reactions. They are used as a marker for clinical diagnosis of peanut allergy in the UK. This study aimed to identify modifications to these allergenic proteins introduced during thermal processing of peanut seeds.

## Methods

Peanuts and derived ingredients were extracted using different buffers and protein composition determined by 2D-PAGE using DiGE with CyDye^TM^. Serum samples from peanut allergic patients were obtained from the Manchester Respiratory and Allergy Biobank and used to assess IgE reactivity to peanuts using immunoblotting.

## Results

Protein solubility was modified by processing and extraction was optimal using harsh denaturing conditions. The 2D protein profiles were reflective of processing type and comparison to raw peanut highlighted spot to spot variations. Roasting caused aggregation leading to smearing but most notable was the apparent loss of proteins including Ara h 1 and Ara h 2/6. Boiling resulted in a loss of proteins into the cooking water and was most extensive in samples boiled for 4-6 hours. Serum IgE from peanut allergic patients bound to Ara h 1, Ara h 3 and aggregates in both roasted and extensively boiled peanuts. In contrast IgE reactivity to Ara h 2/6 in the seeds was lost in boiled peanuts but retained to protein in cooking water.

## Conclusions

Boiling reduces IgE reactivity to peanut seeds, especially using serum samples from patients sensitised predominately to Ara h 2/6. These data further support the premise that boiling may reduce allergenicity of peanuts. The significance of aggregated proteins formed during thermal treatments and their role in IgE binding will be investigated further.

